# Consumers’ Emotion Attitudes towards Organic and Conventional Food: A Comparison Study of Emotional Profiling and Self-Reported Method

**DOI:** 10.3390/foods9010079

**Published:** 2020-01-10

**Authors:** Diana Ismael, Angelika Ploeger

**Affiliations:** Specialized Partnerships in Sustainable Food Systems and Food Sovereignty, University of Kassel, 37213 Kassel, Germany; a.ploeger@uni-kassel.de

**Keywords:** cognitive survey, emotional profiling, food-elicited emotions, food-declared emotions, emotion attitude, color scale, eye-tracking, organic food, conventional food

## Abstract

Emotions represent a major driver behind a consumption behavior. It may provide more important information beyond consumers’ preferences. This study contributes to a better understanding of the discrepancy in emotion attitudes towards organic versus conventional food using a cognitive survey and real food consumption experience. An emotional profiling under informed and uninformed condition, a cognitive survey, and a rapid forced-choice test were carried out with 46 consumers. Our work detected a yawning gap in consumers’ declared emotion attitudes in the cognitive survey and elicited emotion attitudes in the food consumption experience. Results showed that consumers exaggerate their positive emotion attitudes towards organic over conventional and their negative emotion attitudes towards conventional over organic. Even though consumers expressed higher negative emotion attitudes towards conventional food than organic in a cognitive survey, during the emotional profiling they had nearly equal emotion attitudes towards both conventional and organic samples. Moreover, positive declared emotions in a cognitive survey formed a good predictor of the final choice of conventional products over organic under time pressure. However, preferences, declared emotion, as well as elicited emotion attitudes were less useful as predictors of organic choice under time pressure. These results show the importance of taking into consideration the type of applied method when investigating consumers’ emotion attitudes towards organic and conventional products.

## 1. Introduction

We often face a situation where we go to a supermarket thinking about a tasty healthy dinner we want to prepare and having intentions to buy all the ingredients as organic, then, we end up buying less organic than we had planned. Our final behavior could be explained due to the unavailability, high prices, unattractive sensory attributes of the organic product, or simply lacking the emotional motivation.

This situation is acknowledged by researchers as the Intention-Behavior Gap (IBG). IBG denotes a situation in which consumers hold high positive attitudes and intention towards a specific purchase behavior, though their actual behavior falls short to this attitude and intention [1,2,3].

Organic food has been defined by researchers as a food with credence attributes, which are the attributes that cannot be ascertained by the consumer before the purchase or after the consumption [4]. Over the years, consumers have become increasingly concerned with the credence attributes of organic products such as health benefits, environmental-friendliness and animal welfare. Driven by those attributes, the organic food has moved from being just a favored or prestige product to a “must-have” product. Though, the organic food market is usually the most representative market of IBG where consumers usually declare positive attitudes towards organic food, yet they end up purchasing a smaller amount or even not purchasing any [1,3,5,6,7,8].

Most of the theories of IBG in organic food consumption focus on attributing the gap to different barriers such as price, availability, lack of trust, lack of knowledge, habits, and overall liking [3,9,10,11,12]. Nevertheless, the studies that investigate the key role of emotions in the final choice between organic or conventional food are still scarce. Research on food—emotion association report that consumer’s food-related emotions may provide additional information beyond overall liking, and better predict the consumer’s preference and food choice behavior [13,14,15,16,17,18,19,20].

Emotions represent the major driver behind a consumption behavior [21]. Previous studies demonstrate the impact of emotions on organic food choice and acknowledge the importance of several emotions such as pride, guilt, fear, empathy, and disdain in prompting the green consumption behavior [6,22,23,24,25]. Conversely, the influence of food consumption on consumer’s emotions and the relationship of this food–emotion association with regard to food acceptance and preference has only gained attention recently in consumer and sensory research [13,16,21,26,27]. The latter attention has led to the development of various methods to capture consumers’ explicit as well as implicit emotional attitude towards food.

The most common used approach to study explicit food-related emotion attitudes is the self-reported method in which participants declare themselves their food-related emotion attitudes such as interviews, or verbal or nonverbal questionnaires [28]. This method measures the conscious subjective emotion attitudes [29]. On the other hand, the implicit measurements of food-related emotions are indirect and non-self-reported methods and can detect emotions while participants are testing the food without the need of a cognitive translation during or after the testing [20,30,31] such as the color scale method [32].

However, the question is, do the respondents of a self-reported method declare the same attitude they express when going under a real behavioral condition? Researchers observed a gap between the responses declared in a self-reported measure and the detected responses during a given task [2,33,34,35], in particular with attitudes related to “green behavior”. Several studies reported that people may exaggerate their attitudes and tend to appear “greener” than they actually are [2,33]. This can be ascribed to the actual barriers that may control the final behavior or to the social desirability of being seen as a green consumer [36,37,38].

Given the great importance of emotions towards organic and conventional food with respect to final choice, it is crucial to understand the explicit and implicit food-related emotions towards both types of food. A better understanding of consumer’s food-related emotions provides a new way of thinking about the motivational basis of consumers’ behavior, which could be a critical advantage for organic against conventional food choice. 

The presented work hypothesizes that the self-reported measure is a poor predictor of the real consumers’ emotion attitudes towards organic and conventional food. This research, thus, constitutes a relatively new attempt to investigate the emotion attitudes gap between the food-declared emotions in a self-reported questionnaire and the food-elicited emotions detected during an informed and uninformed food consumption experience towards organic and conventional food using explicit and implicit methods. In addition, the study investigates the impact of organic purchase intention and behavior on emotion attitude towards organic food, and vice versa.

Moreover, people tend, under time pressure, to rely on their underlying implicit attitudes to guide their food choices. Time pressure method combined with forced-choice limit the extensive information processing and, thus, decrease the influence of explicit preference and increasing the probability of obtaining more implicit attitudes [2,39,40]. Since some choices at marketplace may be taken under a degree of time pressure, therefore, this study explores the relationship between the consumer’s rapid forced-choice and their preferences, implicit and explicit emotions to understand the effect that those factors may have on consumer’s rapid choice.

## 2. Materials and Methods

**Figure 1:**
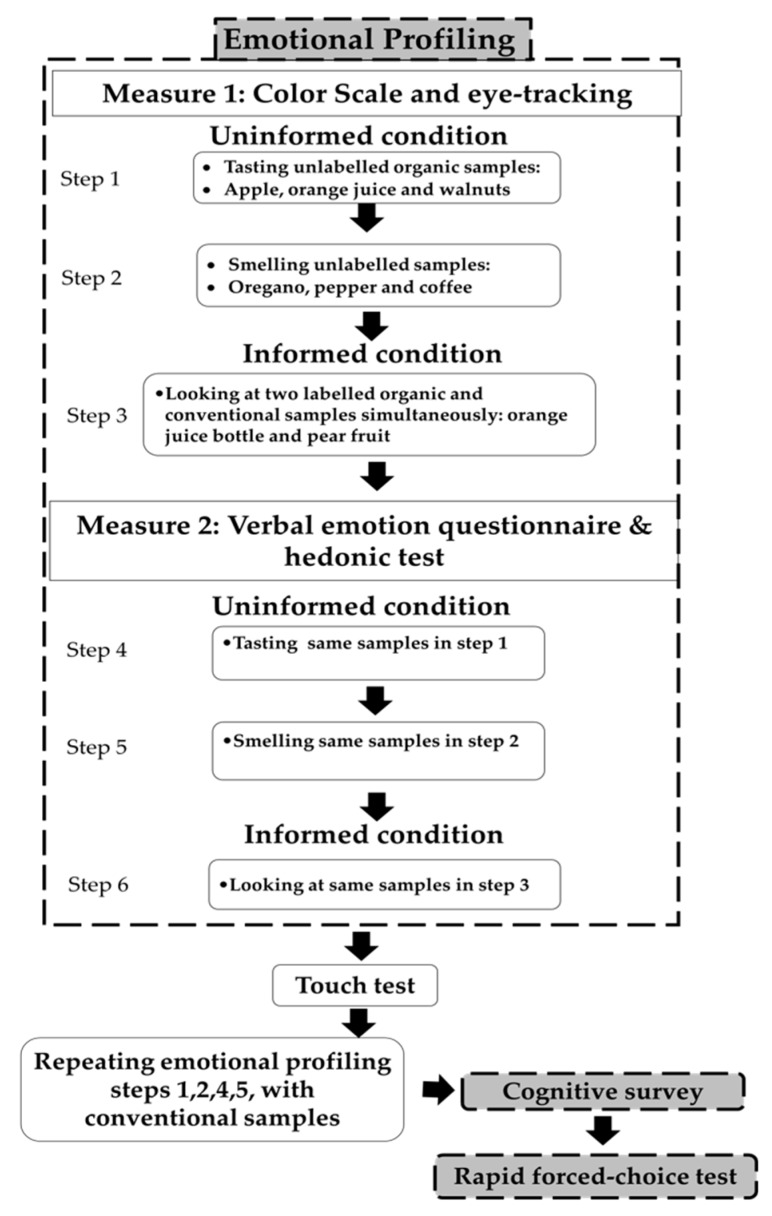
Experimental design that demonstrates the procedures of informed, uninformed emotional profiling, touch test, cognitive survey, and the rapid forced-choice test.

This study employed a self-reported questionnaire (hereafter referred to as the “cognitive survey”) and a consumer test (hereafter referred to as “emotional profiling”) under informed and uninformed conditions in order to investigate consumers’ emotion and attitudes towards organic and conventional food. In addition, a rapid forced-choice test took place as the final step of this experiment. Figure 1 demonstrates the experimental design.

Forty-six consumers volunteered to take part in this research study. Consumers were invited to take part in the experiment throughout invitations sent via emails as well as social media or in person. Each consumer attended individually one experimental session that lasted about 40 min. The experiment took place in standard booths at the sensory facilities of Kassel University and at Fulda University of Applied Sciences.

All individual experiments were carried out in the same booth under the same conditions. None of the consumers reported to have ageusia, anosmia, dyschromatopsia, or color blindness. The difficulty of being eye-tracked was a main criterion of recruiting consumers.

Upon entering the sensory lab, the consumers were thanked and given a short introduction to the sensory facility. A friendly conversation preceded the experiment to relax the participants and release any stress they have that may affect their responses. Subsequently, consumers received a verbal and written introduction about the procedures. However, no additional information was given about the purpose of the experiment.

The emotional profiling preceded the cognitive survey to avoid giving the consumers any prior idea that organic and conventional samples will be used in this experiment. Thus, maintaining their concentration on the food-elicited emotions and overall liking rather than the attempt of realizing the nature of the tested sample (whether organic or conventional produced).

### 2.1. Emotional Profiling

An emotional profiling with food testing (taste, smell, and visual testing) was conducted, considering the conditions of ISO 8589 standards [41] to study consumers’ food-related emotions (hereafter referred to as “food-elicited emotions”) and overall liking towards organic and conventional food samples.

#### 2.1.1. Samples

As shown in Table 1, 21 samples in total were used in this experiment. Out of the 21 samples, 18 were organic and conventional samples that constituted eight food pairs (apple, orange juice, walnut, oregano, red bell pepper, coffee, pear fruit, and orange juice bottles). The food pairs were served in a sequential monadic design. In addition, materials that represent nature (e.g., grass and stones), price (e.g., coins), and health (e.g., apple) aspects of organic products were used in a touch test.

The food samples were chosen to be usually available in the supermarkets and widely used by most of consumers. The names of the samples were announced in the experiment invitation to avoid allergy reaction that consumers may have against any of the samples. Fresh samples were served in room temperature using small glass bowls for apple and walnut samples, a 100 mL glass for processed orange juice, a 250 mL opaque beaker for oregano, red bell pepper, and raw coffee. All consumers received the same portion size of each sample. The samples in the touch test were served in paper envelops, while in the rapid forced-choice test, the samples were placed inside a tuck-in-flap box.

#### 2.1.2. Procedures

The emotional profiling design was developed in the following way. First, taste and smell tests took place respectively in uninformed conditions with unlabeled organic food samples and with no information about the nature of the sample. Next, a visual test in informed conditions took place with a simultaneous serving of organic and conventional labelled samples of the same type. After that, participants underwent a touch test. Then, participants repeated the taste and smell tests with unlabeled conventional samples. The implicit food-elicited emotions were measured first using the color scale with eye-tracking, while the explicit food-elicited emotions were measured after using a verbal emotion questionnaire.

Color scale and eye-tracking measure. The color scale is a new developed method that is used to detect the implicit emotions and consists of two sets of colors: light colors and dark colors. In a previous study [32], it was proven that participants focus on the light colors to express positive emotions and the dark colors to express their negative emotions [32,39]. The eye-tracking data were obtained using an individual laptop equipped with the portable SMI RED-250 mobile eye-tracker powered by USB. The screen-based eye-tracker is manufactured by SensoMotoric Instruments (SMI, Trevor, Germany) and has a sampling rate of 60 Hz. Consumers were provided with instructions on the working mechanism of the eye-tracker.

The emotional profiling was pretested with 13% of the consumers (the pretest data were not included in the final analyzed data) in order to (i) guarantee the optimal experiment design, (ii) define the optimum position of the participant in the booth that allows them to test the sample adequately and at the same time obtain the best results by the eye-tracker, and (iii) avoid boredom by determining the best adequate given time for testing the samples before the automatic appearance of the color scale. A testing period of 20 s among other pre-examined periods by the experimenter (15, 20, 40, and 60 s) was chosen as the optimal period to have an impression (food-elicited emotions) without getting fatigued [30].

At the beginning, participants were seated in the best position that guaranteed the ideal functionality of the eye-tracker. Then, a simulation trial was conducted to familiarize participants with the use of the new color scale and the eye-tracker. Participants were asked to taste, smell, and look at the sample while focusing on their emotions that would be elicited by the sample.

When the sample is being served, an instruction of “Please press Enter when you are ready to test the sample” was displayed on the screen. The consumers then were given a period of 20 s to test the sample, in the meantime, a white screen was being displayed on the laptop. After 20 s, the color scale was automatically displayed on the screen for 5 s. Consumers were instructed to use the color scale to express their food-elicited emotions by fixing their eyes on the color set that expresses the most these emotions.

The light and dark colors position of the color scale was reversed randomly to avoid the bias resulting from presenting the same set of colors on the same side of the screen. To avoid eye-fixation bias that may result from the last eye-fixation tendency, a light-grey cross was shown in the center of the white screen at the end of the 20 s and 2 s prior to the appearance of the color scale [42,43]. After the disappearance of the color scale, consumers signaled to be served the next sample and so forth. Water in room temperature was used as palate cleanser between samples.

Verbal emotion questionnaire. A verbal emotion questionnaire, of 5-point scale (1 = I do not feel it at all, 5 = I strongly feel it) with a rate-all-that-apply (RATA) method, was used in the emotional profiling to detect consumers’ explicit emotions. The verbal emotion questionnaire consisted of 12 emotion words of which six were positive emotion terms (active, satisfied, optimistic, proud, happy, and encouraged) and six were negative emotion terms (guilty, ashamed, angry, sad, regretful, and scared). The order of presenting the emotion terms was randomized among all samples.

After completing the emotional profiling with all samples using the color scale measure, consumers were asked to taste the sample again (if needed) and use the verbal emotion questionnaire to declare their deliberate explicit food-elicited emotions. In addition, they were asked to rate their overall liking using a hedonic scale of 5-point: 1 = “strongly dislike”, 5 = “strongly like”).

Touch test. Organic products are perceived by consumers in different manners. Some consumers perceive it as environmentally friendly products more than healthy products, and vice versa. Others perceived it as a premium priced product. This part intended at investigating the consumers’ perceived aspect of organic products concept by allowing the participants to touch representative materials of each concept. Three groups of representative items were used in this test. The first group represented the environmental-friendliness aspect and contained plant leaves, rough bark, grass, and roses. The second group represented health aspect and contained cotton and apple. The third group contained coins and banknotes that were meant to refer to the consumers’ perception of price premium of organic food products. The materials were put in paper envelops and coded with three-digit codes.

Participants were provided with an explanation of the purpose from each group and instructed to conduct a blind test by touching the items inside the envelops only with their hands without looking inside. They were informed that they should not expect any danger or unpleasant surprises. Then they were asked to rate each representative item “Touch the samples inside each bag from left to right, then rate to what extent each sample represents the way you think of an organic product”.

After this step, participants took a rest (if needed) and repeated the emotional profiling (taste and smell) with conventional food samples (Figure 1).

### 2.2. Cognitive Survey

After the emotional profiling, consumers answered the cognitive survey. The main purpose of the cognitive survey was to investigate the following:

Food-declared emotions (emotion attitude): consumers’ positive and negative emotions (hereafter referred to as “food-declared emotions”) towards organic and conventional food in general were measured using the same verbal emotion questionnaire that was used in the emotional profiling.

Purchase Intention: this part aimed to assess consumers’ willingness to buy organic food with a scale ranging between “I wish to buy all my food products as organic” and “I do not wish to buy any organic food”.

Purchase actual behavior: this part aimed to explore the consumers’ actual purchase behavior of organic food. Consumers were asked to choose one out of five scale-statements ranging between “All my food purchases I currently buy are organic’’ and ‘’ I don’t buy any organic food”.

Organic product concept: this part intended to investigate the consumers’ perceived aspect of organic product concept through self-reporting. Participants were asked to rate three aspects of organic food concept: premium priced products, environmentally friendly products, and health products. The results of this part of the cognitive survey were intended to be compared with the results from the touch test.

Factors influencing organic food consumption behavior: according to previous studies, credence attributes (e.g., health benefits, animal welfare, and environment), in addition to the taste and appearance are among the strongest drivers behind organic food consumption behavior [44,45]. This part aimed to explore the strongest factor that influences the consumer’s organic behavior.

Subjective well-being: the current study investigated the consumers’ perception on the impact of the intention-behavior gap in their organic food consumption on their well-being status [46,47]. This part of the survey started with a short introduction to the common definition of well-being. Then consumers rated their agreements on statements that links their organic food consumption to their well-being status “I believe that consuming organic food is one of the main factors behind having a good well-being”. Finally, consumers were asked to rate their perception on the negative impact that IBG may have on their well-being status “When I go to the market having the intention to buy organic food, but then due to whatever reason I end up not buying it all organic as I wished for. I think this purchasing behavior might have a negative impact on my well-being”. The rating was done using a 5-point agreement scale “1 = strongly disagree, 5 = strongly agree”.

### 2.3. Rapid Forced-Choice Test

As a final step of this experiment, consumers took the rapid forced-choice test under time pressure condition after completing the cognitive survey. Consumers had to make an immediate choice and grab rapidly one of two orange juice bottles (organic and conventional produced) placed inside a closed tuck-in-flap box that was going to be opened for only 2 s “you have only two seconds starting from the moment the box is opened to grab one of whatever exists inside”. Consumers had no clue what was inside the box.

The orange juice bottles placed inside the box were the same bottles served in the informed emotional profiling. Thus, consumers’ overall liking and elicited emotion attitude towards both bottles were previously examined. However, consumers were not informed at any point of the experiment that the rapid test will be conducted with the same bottles. The organic juice bottle was made of cartoon material, while the conventional juice bottle was made of plastic. The two bottles had the same size and almost the same color except for the caps (Appendix A). Their position inside the box was changed randomly among consumers to avoid the bias that may result from presenting each bottle in the same position.

On completion of the experiment, the orange juice bottle that was chosen in the rapid forced-choice was offered to the consumers as a small token of thanks. Consumers had the choice later to change the bottle they grabbed first. However, the data that was considered for analysis was their first choice.

During the whole experiment participants were free to take a short break (3–5 min) between the steps whenever they needed to.

### 2.4. Data Analysis

The collected data was analyzed by using the Statistical Package for Social Sciences (IBM SPSS Statistics version 24, Armonk, NY, USA). First, descriptive statistics were used to summarize the consumers’ demographic characteristics. One participation was excluded as the consumer did not accomplish the simulation trial because of his/her inability to be eye tracked and deliver quality data.

For analytical purposes, positive emotion terms (happy, optimistic, proud, active, satisfied, and encouraged) were computed and considered as the general positive emotions, while the negative emotion terms (sad, guilty, ashamed, regretful, angry, and scared) were considered as the general negative emotions.

The data of the verbal emotion questionnaire were analyzed using paired *t*-test to study the significant differences in general emotions between organic and conventional samples. ANOVA was performed for each sample to investigate the differences in general emotions between genders.

Spearman’s rho test was used to investigate the correlation between overall liking and general positive emotions.

In order to detect any significant differences in dwell time on light colors and dark colors after each organic and conventional sample, the eye-tracking data were analyzed conducting the Wilcoxon signed rank test. In addition, the Wilcoxon signed rank test was used to detect the IBG in consumers’ organic food consumption.

The Mann-Whitney test was used to investigate the differences in the dwell time on the color scale per sample between genders. Besides, the test was used to study the gender impact on purchase intentions and behavior.

The Kruskal–Wallis test was performed to understand the relationship between the patterns of organic food purchase behavior and the start period of consumption.

A generalized linear model was used to explore the correlation between the final choice of organic versus conventional juice bottle and (i) purchase behavior patterns, (ii) overall liking, and (iii) the general positive emotions. In addition, the relationship between consumers intention to buy organic and their positive emotion attitude towards organic was investigated.

## 3. Results

Forty-six consumers aged between 19 and48 years took part in this experiment. Based on the results of the cognitive survey, Table 2 demonstrates the demographic and other characteristics of the consumers. The majority of the consumer sample (96%) had a moderate to very good level of knowledge on organic food. A total of 75% of the consumers were students, 20% were employees, while 5% were neither students nor employees. The results showed no differences between participants’ level of education or occupation on their purchase intentions or behavior.

Environment was the highest rated motivating factor behind organic food consumption. Health was rated after both animal welfare and taste as the fourth driving factor behind organic food consumption.

### 3.1. Food-Elicited Emotions vs. Food-Declared Emotions

#### 3.1.1. Organic vs. Conventional Samples

**Figure 2:**
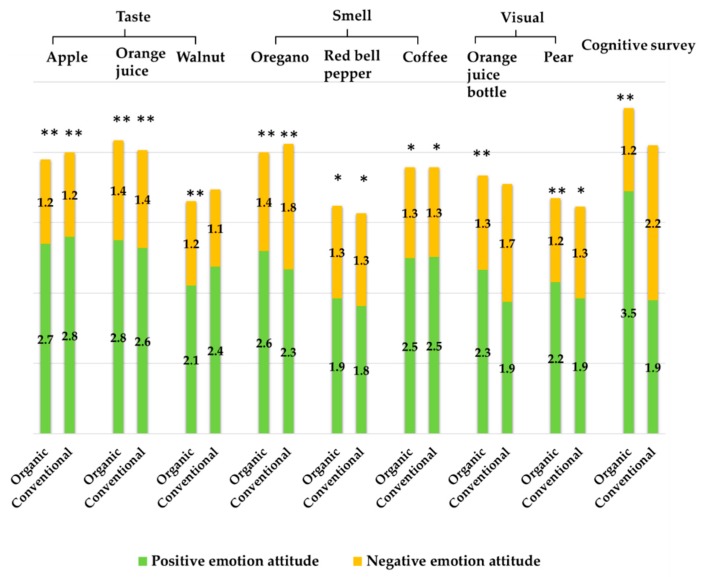
Emotion attitudes: food-elicited emotions expressed in the emotional profiling for each sample, and food-declared emotions declared in the cognitive survey. The data represents the rating values using the verbal emotion questionnaire of a 5-point scale: 1 = I do not feel it at all, 5 = I strongly feel it. (*) Significant differences between positive emotions and negative emotions: * *p* < 0.05, ** *p* < 0.01.

In the cognitive survey, consumers were asked to use the verbal emotion questionnaire to declare their positive food-declared emotions (Dec.PE) and negative food-declared emotions (Dec.NE) towards organic and conventional food in general (Figure 2).

The paired *t*-test showed that Dec.PE towards organic food were significantly higher (*p* < 0.05) than the Dec.PE towards conventional food, while Dec.NE towards conventional food were significantly higher (*p* < 0.05) than towards organic food. In addition, a significant difference (*p* < 0.01) was found between the Dec.PE and the Dec.NE towards organic food (see Appendix B). No significant difference was found between Dec.PE and Dec.NE towards conventional food.

ANOVA detected no gender impact on food-declared emotions towards both organic and conventional food.

In the emotional profiling tests, on the other hand, explicit food-elicited emotions, positive food-elicited emotion (Eli.PE), and negative food-elicited emotions (Eli.NE), towards organic and conventional food samples were measured with the verbal emotion questionnaire. Figure 2 demonstrates Eli.PE and Eli.NE for each sample. The paired *t*-tests resulted in significant differences (*p* < 0.05) between Eli.PE and Eli.NE with all samples except for the conventional orange juice bottle.

In addition, results showed no significant differences neither in positive nor negative emotions between organic and conventional samples except for oregano and orange juice bottle. Consumers rated their Eli.PE towards organic orange juice bottle and oregano samples higher than the conventional samples, while they rated their Eli.NE towards conventional orange juice bottle and oregano samples higher than the organic samples (see Appendix B).

ANOVA showed no gender impact on food-elicited emotions towards organic and conventional samples.

Table 3 displays the highest three rated emotion terms that were used by the consumers to express their food-elicited and food-declared emotions. Happy, optimistic, and active were the highest rated emotion terms used in the cognitive survey to declare consumers’ emotions towards organic food. Guilty, regretful, and happy were the highest used terms to declare emotions towards conventional food. Similar to the cognitive survey, happy, active, and optimistic were the most used and highest rated terms to express food-elicited emotions for all samples in the emotional profiling. Yet, guilty, sad, and happy were the most used terms to express emotions towards the conventional orange juice bottle.

A new developed color scale, combined with an eye-tracking tool under time pressure condition, was used in the emotional profiling to detect implicit food-elicited emotions. Participants were asked to use the color scale displayed on the screen (for 5 s) and focus on the color set that expresses their food-elicited emotions after testing the sample. Dwell time on each of the light and the dark colors was calculated. Participants usually use the light colors to express implicit positive emotions, while they use the dark colors to express implicit negative emotions [32]. Similar to the results of the verbal emotion questionnaire, the Wilcoxon signed rank test showed no significant differences in implicit food-elicited emotions (*p* < 0.05) between organic and conventional samples.

**Figure 3:**
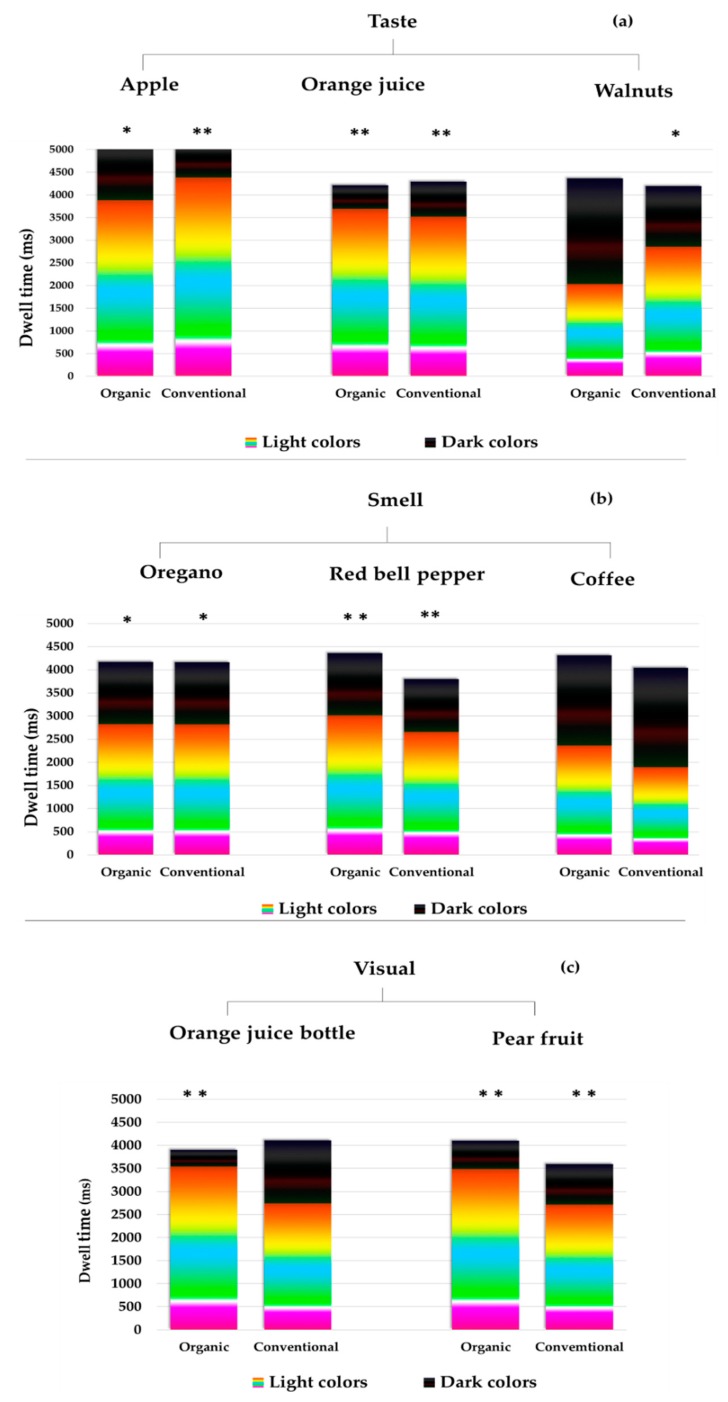
Dwell time in milliseconds on light colors and dark colors to express implicit food-elicited emotions towards organic and conventional samples in the emotional profiling using the color scale. The display time of the color scale on the screen lasted for 5000 ms. (*) Significant differences in dwell time between light colors and dark colors (* *p* < 0.05, ** *p* < 0.01). (**a**) The results of the taste test, (**b**) smell test, and (**c**) visual test.

Significant differences in dwell time between light colors and dark colors were found for all samples except for the organic walnut, organic, and conventional coffee and conventional orange juice bottle. Figure 3a–c elucidates the average dwell time on each set of colors in the color scale.

The Mann–Whitney test showed a significant difference between males and females in dwell time on dark colors after tasting organic walnuts, where women had higher dwell time (2856 ms) on the dark colors than men (1345 ms), and smelling organic red bell pepper, where men had higher dwell time (2190 ms) on the dark colors than women (889 ms).

#### 3.1.2. Cognitive Survey vs. Emotional Profiling

In the cognitive survey consumers rated their Dec.NE of conventional food higher than Dec.PE, while in the emotional profiling their Eli.NE of all conventional samples were always rated lower than Eli.PE.

When comparing the emotions between the cognitive survey and the emotional profiling towards the conventional food/sample, Dec.NE towards conventional food in the cognitive survey were significantly higher (*p* < 0.05) than Eli.NE towards all conventional samples in the emotional profiling. In contrast, consumers’ Dec.PE towards conventional food in the cognitive survey were significantly lower (*p* < 0.05) than their Eli.PE towards conventional samples in the uninformed emotional profiling, except for red bell pepper. However, Dec.PE for conventional food in the cognitive survey were not significantly different from the Eli.PE towards the conventional samples in the informed emotional profiling.

In terms of organic samples, positive elicited emotions towards all organic samples in the emotional profiling were significantly lower (*p* < 0.05) than the declared positive emotions towards organic food in the cognitive survey. Nevertheless, there were no significant differences in negative emotions between the cognitive survey and the emotional profiling except for orange juice sample in the taste test.

### 3.2. Consumers’ Preferences: Organic vs. Conventional Food

**Figure 4:**
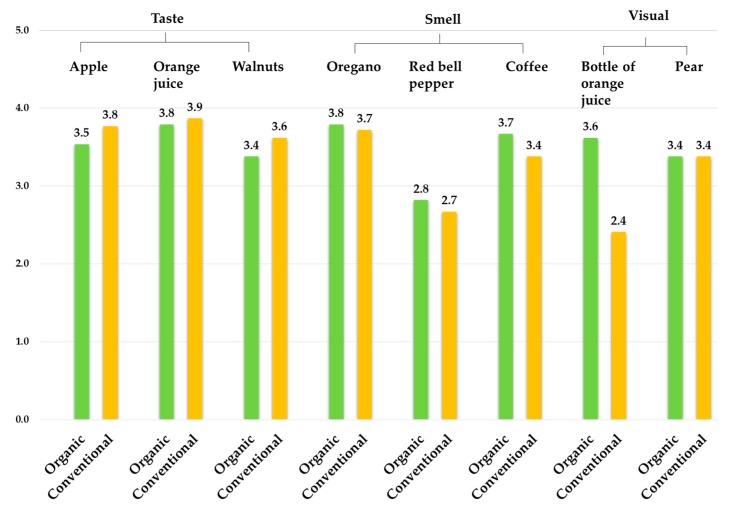
Overall liking mean values for each of the sample (organic and conventional products) on a 5-point scale: 1 = I strongly dislike, 5 = I strongly like.

As shown in Figure 4, no significant differences in overall liking between organic and conventional samples were found with all the samples except for the orange juice bottles. The taste of conventional samples was slightly more liked by consumers than the taste of the organic ones, while the smell of the organic samples was slightly more liked than the conventional ones. In the informed emotional profiling, consumers liked the organic orange juice bottle significantly more than the conventional juice bottle (*p* < 0.05), while they liked both the organic and conventional pear fruits almost the same.

Furthermore, as shown in Table 4, Spearman’s correlation test revealed a significant moderate to a very strong correlation between overall liking and positive elicited emotions for each of the samples. The high rates of positive emotions were associated with higher rates of overall liking.

### 3.3. Organic Food Concept: Cognitive Survey vs. Touch Test

In the cognitive survey, the organic product was perceived more as an environmentally friendly product, than as a healthy product, and finally as an expensive product. Results from touch test were consistent with regards to the environmentally friendly aspect, while premium price and health aspects differed but still had very close rate values.

**Figure 5:**
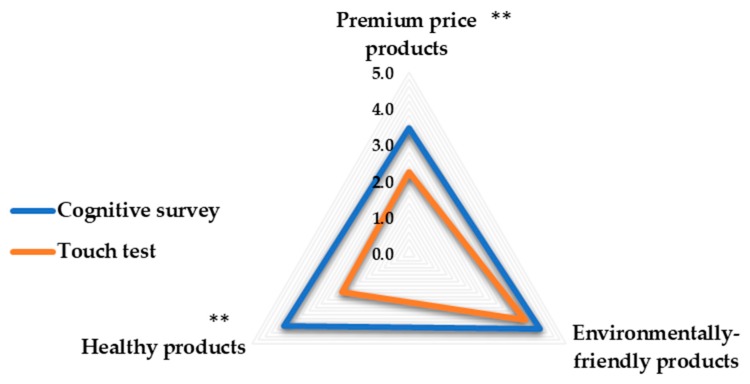
Spider diagram showing the differences in participants’ perceived aspect of organic product concept between the cognitive survey and the touch test. The rating was done on a 5-point scale: 1 = it does not represent the concept of the organic product at all, 5 = it highly represents the concept of the organic product. (*) Significant difference in the responses between the cognitive survey and touch test, ** *p* < 0.01.

Figure 5 illustrates the comparable results between the participants’ perception of the three mentioned aspects. Significant differences between the responses in the cognitive survey and touch test were found in premium price and health aspects. Participants rated those two aspects in the cognitive survey higher than in the touch test.

### 3.4. Intention-Behavior Gap (IBG)

**Figure 6:**
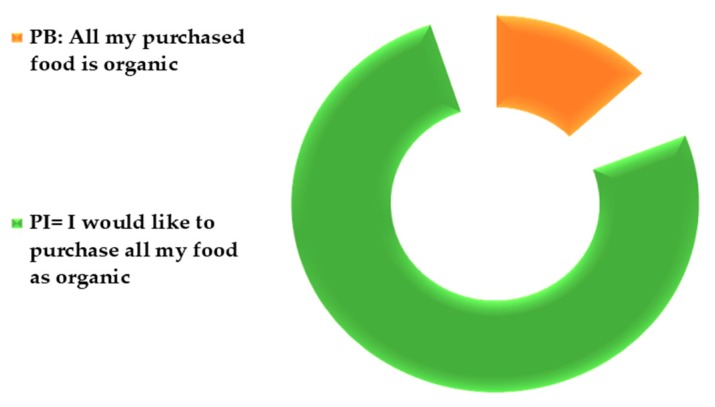
The percentage of consumers who held intentions to buy all their food as organic (PI: purchase intentions) versus the percentage of consumers who reported to purchase all their food as organic (PB: purchase behavior).

As shown in Table 2, 76% of the consumers held high intentions to buy all their food purchases as organic. Yet, only 13% of the consumers stated that they buy all their food purchases as organic (Figure 6).

**Figure 7:**
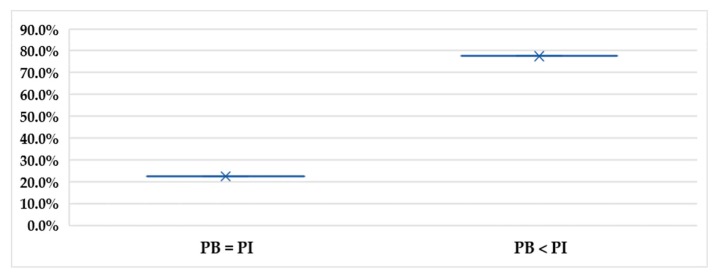
The percentage of the consumers who hold purchase intentions (PI) equal to their purchase behavior (PB), and consumers who hold PI higher than their PB.

Figure 7 illustrates the personal intention-behavior gap where 77.5% of the consumers have a purchase behavior less than their declared intentions, while 22.5% of the consumers purchase the same amount as they intend to do. The Wilcoxon signed rank test found a significant difference (*p* < 0.01) between consumers’ intention to buy organic food and their real purchase behavior.

No significant impact of purchase behavior or purchase intention was found on the declared positive emotions towards organic and conventional food. On the other hand, results indicated that positive emotion attitudes towards organic and conventional food is not a significant predictor of neither organic food purchase intention nor behavior.

The Mann–Whitney analysis showed no gender impact on the purchase behavior or purchase intention (*p* > 0.05).

#### Perceived Impact of IBG on Subjective Well-Being

Seventy-five percent of the consumers were satisfied with their general well-being state, half of this percentage believed that organic food consumption had the main impact on their good well-being state.

When consumers were asked about the perceived influence of the intention-behavior gap in their organic food consumption on the subjective well-being, 22% agreed on the negative impact of IBG on their well-being state, while half of the consumers believed that the IBG had no influence on their well-being. In addition, all consumers who agreed on the impact of the organic behavior on their good well-being had a high consumption behavior (they purchase more than a half of their food as organic).

### 3.5. Rapid Forced-Choice

In the rapid forced-choice test, the participants had 2 s to choose one of two orange juice bottles (organic and conventional) that were put in a closed box. Forty percent of the participants chose the conventional orange juice bottle. Among the participants who had higher positive emotions towards organic orange juice bottle in the emotional profiling, 55% chose eventually the conventional juice bottle. Moreover, 57% of the participants who liked the organic juice bottle more than the conventional one chose the conventional juice bottle in rapid forced-choice test.

A generalized linear model was used to assess the degrees of impact of (i) overall liking, (ii) Eli.PE in the emotional profiling, (iii) Dec.PE in the cognitive survey, and (iv) purchase behavior patterns on the final choice of organic versus conventional juice bottle.

Overall liking and Eli.PE towards organic juice bottle were poor yet significant predictors (*p* < 0.1) of the final choice for organic juice bottle under time pressure. The participants who showed higher positive emotion attitudes and had stronger preference towards the organic juice bottle tended eventually to choose the conventional bottle.

On the other hand, Dec.PE towards conventional food in the cognitive survey was found as a significant good predictor of the final choice of conventional bottle in comparison with the organic one. The participants who declared more positive emotions towards conventional food in the cognitive survey, tended to choose the conventional juice bottle at the end.

The study found that neither participants’ reported intention nor behavior patterns were significant predictors of their rapid choice of organic versus conventional juice bottle.

## 4. Discussion

To investigate the differences in people’s emotion attitudes towards organic and conventional food/samples, this study applied three methods: a cognitive survey and an emotional profiling under two types of conditions: informed and uninformed conditions, with explicit and implicit measures. In this study, we differentiate between food-declared emotions that are declared in the cognitive survey and food-elicited emotions that are expressed in the emotional profiling.

Knowledge on organic food concept was an important aspect to be considered when recruiting consumers. Notwithstanding, recruiting consumers was random, 96% of the consumer sample had moderate to very good knowledge on organic food concept. This could be explained by the educational level the consumers had as most of the consumers were still students or held a university degree. An additional explanation could be the growing interest in the benefits of the organic produced food as well as people’s increased concerns about health issues, environment, and other aspects related to the sustainability concept.

Taste, as mentioned in previous research [45,48], is considered one of the most important factors that influence organic consumption. In this study, consumers rated the factor “taste” as one of the first three factors behind their organic consumption. They believed that organic food has better taste than conventional food. Yet, consumers equally liked the taste of conventional samples as organic ones in the uninformed test, which is consistent with other works [49,50]. This shows how using a self-reported method to assess a certain sensory attribute of an organic product may results in an outcome that diverges from the outcome of a real consumption experience.

Moreover, consuming organic food since childhood had a relationship, yet not significant, with current organic purchase behavior. This shows the important role of childhood food habits that influence the adult food behavior [51].

### 4.1. Food-Declared Emotions vs. Food-Elicited Emotions

#### 4.1.1. Organic versus Conventional

In the cognitive survey, consumers rated their positive food-declared emotions towards organic food significantly higher than the conventional food and declared significant higher negative emotions towards conventional than organic food. Likewise, the results of the informed emotional profiling of organic and conventional orange juice bottles were consistent with the cognitive survey. However, the results obtained from the uninformed emotional profiling were different. Neither explicit nor implicit measures detected any significant differences in food-elicited emotions between organic and conventional samples. This shows how consumers’ emotional attitudes may change between informed and uninformed tests. The results also suggest that in a cognitive survey and an emotional profiling under informed condition, consumers would exaggerate their positive emotion attitudes towards organic over conventional food/samples, and their negative emotion attitudes towards conventional over organic food/samples. This exaggeration could be largely guided by the perceived credence attributes of organic over conventional food [48]. The word (or the label) “Organic/Bio” might have an impact on both declared and elicited emotion attitudes. These results were in line with previous studies that showed that once a product was labelled or referred to as organic it would trigger higher positive attitudes or willingness-to-pay for this product [18,48,52,53,54,55,56].

This could be explained due to the cognitive bias, which is a factor that affects people’s judgment and interpretation of their attitudes. Throughout the years, the credence benefits of organic food were overstated by marketing strategies. The ongoing advertisements promote organic food as a credence product that holds advantages to the human health, animal welfare, and the environment. The emphasizing on the organic food advantages versus the disadvantages of the conventional food led the consumers to shape a stereotype of those two categories of products. Consumers in this study may have been affected by the mentioned stereotype, and thus made the assessment of their emotion attitudes towards organic and conventional food in the cognitive survey and the informed consumption experience based on the preconceived credence attributes they usually associate with organic food.

However, the focus of the market on organic as a credence attribute is still not enough to boost consumers’ purchases behavior and narrow the consumers’ IBG that was demonstrated in this study.

#### 4.1.2. Cognitive Survey versus Emotional Profiling

The results obtained from the comparison between the cognitive survey and the emotional profiling revealed two important points.

First, consumers would declare higher negative attitudes towards conventional food in a cognitive survey than their expressed attitudes in both informed and uninformed consumption experience. Our results confirm previous findings on how consumers may change their detected attitudes with different methods [2,33,34,35,57].

While consumers’ Eli.PE towards conventional samples in the uninformed emotional profiling (taste and smell) were higher than their Dec.PE in the cognitive survey, their declared and elicited emotions were not significantly different between the cognitive survey and the informed emotional profiling (visual test). From these results, it is clear that consumers’ positive emotion attitudes would be changed when they know for certain that they are testing a conventional sample against an organic one. This result ties well with previous studies [53,58] wherein consumers judged a product due to a preconceived notion or opinion even before testing it.

Secondly, people would overstate their positive emotion attitudes and they would understate their negative emotion attitudes towards organic food in a cognitive survey compared with a real consumption experience.

The different outcomes between the cognitive survey and the emotional profiling obtained in this study could have resulted from the sample types. Though, our study suggests taking into consideration the effect of the different experimental methods (self-report method and emotional profiling in informed and uninformed conditions). The significant differences in attitudes, which were evident when the label “Organic/Bio” was visible, may have resulted due to the different applied methods.

Table 5 demonstrates the strength of each method in predicting emotion attitudes towards organic and conventional food/samples. Each of the cognitive surveys, informed and uninformed emotional profiling represented good methods (+) of reporting the positive and negative emotion attitudes towards organic food/samples. Both the cognitive survey and informed emotional profiling were not strong methods (−) in reporting the positive or negative emotion attitudes towards conventional food. Consumers’ declared attitudes were affected by the comparison between the organic and conventional food.

However, the uninformed method was considered the strongest (++) among the other methods in reporting positive and negative emotion attitudes towards organic and conventional samples as consumers were not influenced by the organic label impact. Thus, their attitudes were not based on the comparison between the nature of the samples (organic and conventional). Food-declared emotions in the cognitive survey towards organic food were not predictors (−) of the final choice of organic sample under time pressure. Moreover, Eli.PE and preferences in the emotional profiling towards organic were significant but negative predictors (− −) of the final choice of organic (see Section 3.5). On the other hand, declared emotion attitudes towards conventional food represented a significant predictor (++) of the final choice of the conventional sample.

The terms guilty and happy were the most associated emotion terms with the conventional concept by consumers in both the cognitive survey and informed emotional profiling. Similar findings related to the feeling of guilt were reported by previous studies [22,23]. These results go beyond previous reports, showing that the consumers also used the term “happy” to express their food-declared and food-elicited emotions towards conventional food or samples.

Beside focusing on the credence benefits of organic food, the organic marketing strategies is emphasizing the negative impacts of the conventional food production method on the different aspects of life. People would usually feel positive emotions when consuming food, yet their responsibilities towards their health, environment, and animal welfare would lead to mix the feeling of pleasure to have food with the feeling of guilt to have conventional food in particular.

The present study confirmed the findings about the strong correlation between positive food-elicited emotions and overall liking [20]. Higher rates of positive emotions were accompanied with higher rates of overall liking for all samples and vice versa. For red bell pepper, the low rate of overall liking was consistent with lower rate of positive emotions.

#### 4.1.3. Organic Food Concept: Cognitive Survey vs. Touch Test

The results from the touch test also showed the differences between the responses obtained from a cognitive survey and the responses obtained from a real touch experience. Health was perceived more related to organic concept than premium price in the cognitive survey, while in the touch, those aspects were almost perceived the same. Yet, premium price and health aspects were rated higher in the cognitive survey than the touch test.

### 4.2. Intention-Behavior Gap

An intention-behavior gap appeared clearly among the consumers in their organic food purchases. Most of the consumers reported an organic purchase behavior that is less than their intentions. Nonetheless, some consumers are quite satisfied with their organic consumption pattern, and their behavior meets their intentions. Reasons behind this gap were not discussed in this study, as it is planned to be discussed in further related work. However, according to previous research, price is the major barrier behind the intention-behavior gap [3].

Similar to the results that were found in the rapid forced-choice test on the impact of consumers’ Dec.PE on their organic choice in the lab, Dec.PE towards organic samples were not significant predictors of their daily life organic food purchase patterns. Besides, the reported purchase intentions and behavior had no relationship with the declared organic emotion attitudes. Nevertheless, data showed that the higher positive attitudes consumers hold towards organic food, the more they buy or wish to buy. Additionally, the study found that neither gender, nor time of starting organic consumption had a significant influence on consumers’ organic purchase behavior. This suggests that daily life purchasing of organic food could be subject to other factors than the ones that were studied in this work.

### 4.3. Rapid Forced-Choice

This research intended to use the time pressure condition combined with forced-choice to reduce the conscious impact on the preferred choice and avoid deliberate decision [34,59].

In the rapid forced choice test, participants had to rapidly choose one of two orange juice bottles (organic and conventional). The two bottles differed in shape and materials and were similar in size and color. The label ‘’Bio’’ was distinct and readable on the organic juice bottle.

As mentioned before, participants were not informed at any point during the experiment about the content of the box in the rapid test. Yet, during the informed emotional profiling that preceded the rapid test, participants had enough time to check both bottles, discern the organic from the conventional one, and evaluate their implicit and explicit emotions with overall liking towards each bottle.

Under time pressure, nearly half of the participants were more likely to choose the conventional juice bottle. Over half of the participants who had higher preference and higher positive attitudes towards the organic juice bottle in the emotional profiling, tended to choose the conventional bottle eventually.

Positive declared emotion attitudes in the cognitive survey form a good predictor of the final choice of conventional bottle, while they did not represent a good predictor of organic final choice. Our study suggests that the likelihood that consumers will choose a conventional orange juice bottle over the organic one under time pressure could be predicted by their positive emotion attitudes towards conventional in a self-reported method. However, the consumers’ choice of the organic bottle under time pressure was unable to be predicted by any of the factors related to the participants’ preferences, declared emotions, elicited emotions as well as their daily lives purchase intentions and behavior.

It was proven that people’s behavior under time pressure is intuitive, guided by their underlying implicit attitudes. Time pressure bounds the information processing and decreases the influence of personal preferences on the final choice [34,39,40,59]. This suggests that the consumers’ underlying choice of the conventional juice bottle over the organic one was led by one or more of different implicit factors such as the trade-off between organic and conventional products in everyday life like price and availability, or simply the material, shape, or position of the bottle inside the box.

Regardless of the position inside the box, all the other factors that have driven the participants to choose the conventional juice bottle over the organic one are considered as advantages in favor of the conventional juice.

In other words, whatever the factor that drove the participants rapid choice, this final choice reflects the perceived advantages of the conventional juice over the organic one. These advantages may have led to an underlying preference of the conventional juice, notwithstanding the participants’ high declared positive emotions and overall liking for the organic juice vs. the conventional juice. This may have an implication in real life shopping at the supermarket as consumers sometimes try to reduce the time spent on grocery food, and thus do the shopping in some cases under time pressure.

### 4.4. Limitations of the Study

The findings of this study should be considered in light of some limitations. First, the data of the cognitive survey was obtained from a small sample size. Besides, the majority of the consumers were students, the thing that makes the sample lack population representativeness. Thus, the results cannot be generalized. However, this research study was the first, to our best knowledge, to investigate the differences in consumers’ emotion attitude towards organic and conventional food applying three methods. The focus was to understand which method is able to detect the best consumers’ real emotion attitudes.

It is advisable to do this comparison study of the three applied methods (informed and uniformed emotional profiling and the cognitive survey) with a larger sample size in order to investigate more predictors of the consumers’ underlying preferences and investigate the impact of the different segments (e.g. occupation, education, and age) of consumers on their real emotion attitudes towards organic vs. conventional food or sample.

The “Bio” label that refers to the organic nature of the pear sample was not evident on the fruit throughout the experiment. Most of the consumers were not able to differentiate between the organic and the conventional fruit. This led to having the same results of overall liking and general emotions for both fruits, which was similar to the other samples from the uniformed tests. Thus, the informed emotional profiling was considered to be conducted with only one pair of samples. In addition, the rapid forced-choice test was also carried out with only one pair of samples. More samples are recommended to be used in both tests.

Despite this limitation, the results of this study have an important implication for future research efforts. The results may suggest that self-reported methods and informed emotional profiling are not the perfect methods to measure the real emotion attitudes for organic vs. conventional food.

## 5. Conclusions

No matter how consumers in this study expressed higher negative emotion attitudes towards conventional than organic food in the cognitive survey, they would still rate their emotions towards conventional food almost equally to organic food, or maybe higher, in a food consumption experience.

This paper argued that cognitive (self-reported) methods are not a good representation of real emotion attitudes towards organic versus conventional food. Consumers tended to appear as holding more positive emotions towards organic food and more negative emotions towards conventional food in self-reported method. However, in the emotional profiling, which assimilates a real food consumption, consumers seemed not to have any different emotion attitudes for organic and conventional food.

Moreover, when consumers declared positive emotions towards conventional food in the cognitive survey, this declaration could predict their final behavior of conventional food choice in the presence of organic choice under time pressure. Yet, this did not apply to the organic sample.

On the other hand, when consumers underwent a situation where they had to express their attitudes towards organic versus conventional samples, neither their implicit nor explicit emotions together with preferences would perform as good predictors of their final choice of organic vs. conventional sample. Apparently, consumers were affected by the “Organic/Bio” label and tended to overstate their overall linking and positive emotion attitudes towards organic over conventional sample, which might have resulted into a misinterpretation of the final choice between organic and conventional sample under time pressure.

In summary, this study detected a yawning gap in people’s emotion attitudes between cognitive survey and food consumption experience under informed and uninformed conditions. This gap is considered a disadvantage in organic food marketing as it may lead to misunderstanding the consumer behavior.

## Figures and Tables

**Table 1:** Type and specification of the samples that were used in the emotional profiling.

	Sample Type and Description			
Taste	Unlabeled sample	Apple	Orange juice	Walnut
		Fresh fruit (Royal gala)	Processed juice	Raw, unsalted
Smell	Unlabeled sample	Red bell pepper	Oregano	Ground coffee
		Fresh	Spices	Valve-sealed bags
Visual	Labeled sample	Pear	Orange juice	
		Fresh fruit (Abate Fetel)	33cl bottle	
Touch	Sample	Coins and banknotes	Fresh grass, stones, tree branches, and soil	Apple and cotton wool balls
	Indication	Indicates the price value of organic product	Indicates the environmental friendliness value	Indicates the health value
Rapid forced-choice	Labeled sample	A 33cl bottle of Orange juice		

**Table 2:** Consumers’ demographic characteristics, organic purchase intention and behavior, in addition to the motivating factors behind the organic food purchasing. The data was collected from the cognitive survey.

Demographic Characteristics	Category	Percentage/Mean
Gender (%)	Female	65%
	Male	35%
Education (%)	University level	75%
	High school	25%
	Less than high school	0%
Occupation (%)	Student	75%
	Employee	20%
	Neither	5%
Knowledge of organic food concept (%)	Very good knowledge	15%
	Good knowledge	58%
	Moderate knowledge	23%
	Poor knowledge	3%
	Very poor knowledge	3%
Organic food consumption behavior (%)	All the food purchases are organic	13%
	Most of the food purchases are organic	40%
	Half of the food purchases are organic	28%
	Only few of the food purchases are organic	18%
	None of the food purchases are organic	3%
Organic food consumption intention (%)	I wish to buy all food purchases as organic	76%
	I wish to buy most of the food purchases as organic	10%
	I wish to buy half of the food purchases as organic	5%
	I wish to buy only few of the food purchases as organic	8%
	I do not wish to buy any organic food	3%
Started consuming organic food (%)	Recently (<5 years)	18%
	Since few years (>5 years)	54%
	Since I was a child	28%
Motivating factors influencing the organic food consumption behavior (5-point scale)	Protecting the environment	4.72
	Animal welfare	4.33
	Taste	3.90
	Health	3.75
	Appearance	2.77
	Rewarding yourself	2.55
Perceived aspect of organic food products (5-point scale)	Environmentally friendly products	4.18
	Healthy products	3.98
	High price products	3.45

Data showed no significant relationship between organic food purchase patterns and the starting period of organic food consumption (recently, few years ago, or since childhood). Yet, 82% of consumers who started consuming organic food since childhood stated that most to all of their food purchases are organic. In addition, the Mann-Whitney test showed no gender impact on consumers’ purchase behavior or purchase intentions.

**Table 3:** Mean values (± SD) of the highest three rated emotion terms (active, satisfied, optimistic, proud, happy, encouraged, guilty, ashamed, angry, sad, regretful, scared, and angry), on a 5-point scale (1 = I do not feel it at all, 5 = I strongly feel it), that were used to express the food-elicited emotions in the emotional profiling and the food-declared emotions in the cognitive survey.

Samples		Emotion 1	Mean	± SD	Emotion 2	Mean	± SD	Emotion 3	Mean	± SD
Apple	Organic	Happy	3.23	1.37	Active	2.90	1.31	Optimistic	2.85	1.39
	Conventional	Happy	3.46	1.23	Optimistic	2.85	1.33	Active	2.79	1.44
Orange juice	Organic	Active	3.28	1.32	Happy	3.13	1.32	Optimistic	2.82	1.35
	Conventional	Happy	3.51	1.39	Optimistic	3.08	1.35	Active	2.90	1.35
Walnut	Organic	Happy	3.59	1.50	Active	2.21	1.30	Optimistic	2.26	1.37
	Conventional	Happy	2.85	1.51	Optimistic	2.59	1.52	Active marketplace	2.26	1.31
Oregano	Organic	Optimistic	2.85	1.33	Happy	2.82	1.45	Active	2.51	1.30
	Conventional	Happy	3.13	1.49	Optimistic	2.97	1.42	Active	2.87	1.36
Red bel pepper	Organic	Active	2.21	1.26	Happy	2.18	1.27	Optimistic	2.15	1.31
	Conventional	Active	2.10	1.12	Optimistic	2.00	1.08	Happy	1.95	1.07
Coffee	Organic	Happy	2.95	1.62	Optimistic	2.90	1.55	Encouraged	2.79	1.63
	Conventional	Happy	3.03	1.56	Optimistic	2.74	1.50	Active	2.74	1.58
Pear fruit	Organic	Happy	2.49	1.34	Optimistic	2.59	1.29	Active	2.36	1.20
	Conventional	Happy	2.29	1.23	Optimistic	2.23	1.27	Active	2.03	1.27
Orange juice bottle	Organic	Happy	2.95	1.52	Optimistic	2.59	1.31	Satisfied	2.51	1.20
	Conventional	Guilty	2.00	1.32	Sad	1.97	1.27	Happy	2.00	1.05
Cognitive survey	Organic	Happy	4.00	1.11	Optimistic	3.78	1.17	Active	3.40	1.00
	Conventional	Guilty	2.60	1.31	Regretful	2.47	1.18	Happy	2.75	1.10

**Table 4:** Spearman’s correlation measures the strength of association between overall liking and positive elicited emotions.

	Overall Liking								
**Positive elicited emotions**	Sample	Apple	Orange	Walnut	Oregano	Pepper	Coffee	Juice bottle	Pear fruit
	Organic	0.661 **	0.641 **	0.619 **	0.719 **	0.769 **	0.696 **	0.595 **	0.606 **
	Conventional	0.812 **	0.438 **	0.682 **	0.658 **	0.623**	0.707 **	0.581 **	0.448 **

** *p* < 0.05.

**Table 5:** The strength of the different applied methods in predicting emotion attitudes and final choice behavior of organic vs. conventional food or samples. (*) represents the significant results.

	Detect Real PEA towards Organic	Detect Real NEA towards Organic	Detect Real PEA towards Conventional	Detect Real NEA towards Conventional	Predict the Final Choice of Organic	Predict the Final Choice of Conventional
Cognitive survey	+	+	−	−	− − (*)	++ (*)
Emotional profiling (Informed condition)			+	+	− −	−
Emotional profiling (Uninformed condition)	++	++	++	++		

PEA: positive emotion attitude. NEA: negative emotion attitude.

**Figure A1:**
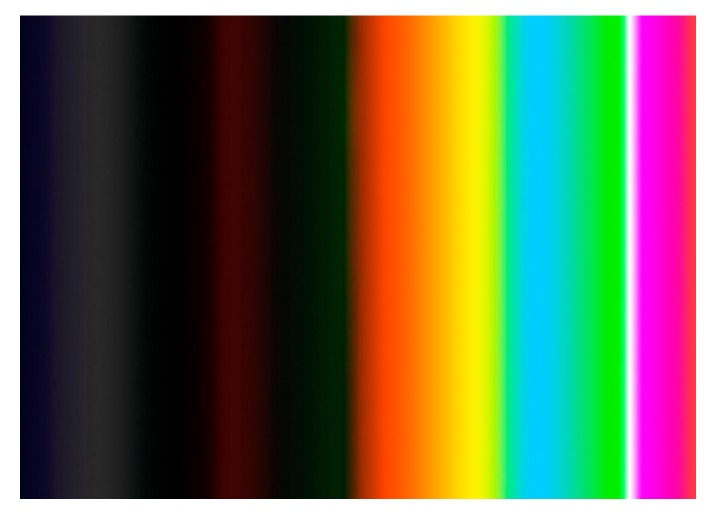
The color scale.

**Figure A2:**
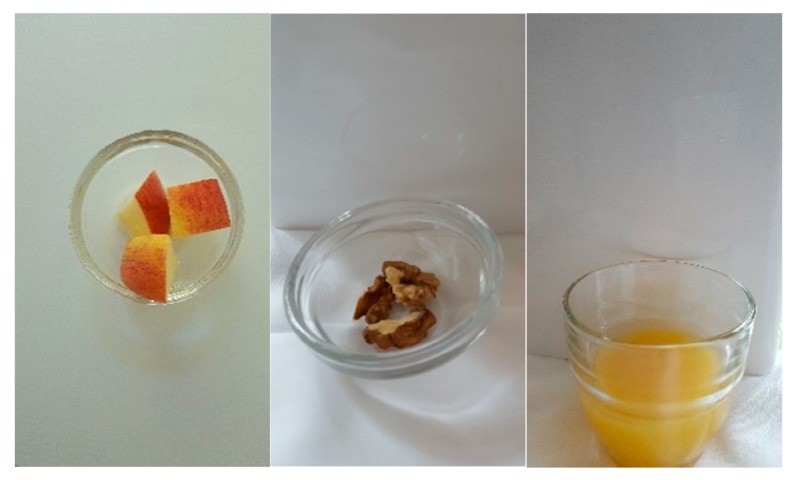
The served samples in the emotional profiling (taste test).

**Figure A3:**
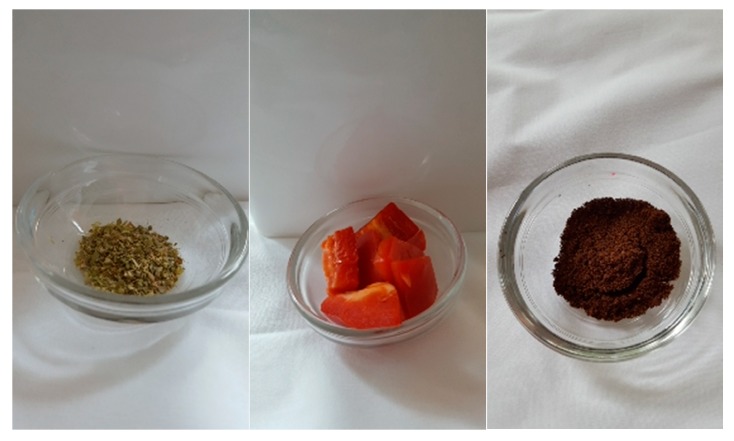
The served samples in the emotional profiling (smell test).

**Figure A4:**
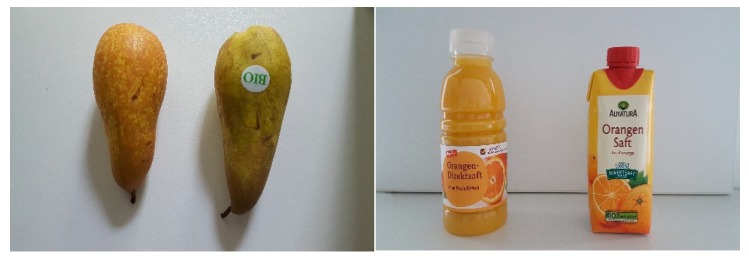
The served samples in the emotional profiling (visual test).

## Appendix B

**Table A1:** Means and significant values of food-elicited emotion, food-declared emotions, overall liking, and dwell time on the color scale.

Sample	Type of Samples	PE	*p* Value Or-Co	NE	*p* Value Or-Co	*p* Value PE-NE	Overall Liking	*p* Value Or-Co	Dwell Time LC	*p* Value Or-Co	Dwell Time DC	*p* Value Or-Co	*p* Value LC-DC
Apple	Or	2.70	0.456	1.20	0.789	0.000	3.54	0.228	3886.80	0.324	1367.89	0.324	0.014
	Co	2.80		1.20		0.000	3.77		4383.20		827.66		0.000
Orange	Or	2.75	0.590	1.42	0.203	0.000	3.79	0.795	3517.56	0.665	520.60	0.168	0.000
	Co	2.64		1.39		0.000	3.87		3302.96		777.00		0.000
Walnut	Or	2.11	0.082	1.19	0.084	0.000	3.38	0.309	2032.90	0.019	2327.04	0.004	0.548
	Co	2.40		1.09		0.000	3.62		2853.31		1339.43		0.025
Oregano	Or	2.60	0.027	1.40	0.000	0.000	3.79	0.562	2825.56	0.77	1348.09	0.55	0.001
	Co	2.34		1.78		0.000	3.72		2824.23		1343.61		0.03
Red pepper	Or	1.93	0.380	1.32	0.993	0.003	2.82	0.411	3016.57	0.460	1344.77	0.827	0.006
	Co	1.82		1.32		0.008	2.67		2658.45		1143.61		0.005
Coffee	Or	2.50	0.900	1.29	0.756	0.000	3.67	0.106	2363.26	0.301	1951.30	0.655	0.3
	Co	2.51		1.27		0.000	3.38		1898.78		2145.97		0.63
Orange juice	Or	2.33	0.018	1.34	0.029	0.000	3.8	0.000	3536.48	0.076	368.23	0.002	0.000
	Co	1.88		1.68		0.368	2.41		2746.32		1359.26		0.058
Pear	Or	2.15	0.058	1.20	0.253	0.000	3.5	0.970	3486.41	0.002	609.91	0.102	0.000
	Co	1.93		1.30		0.002	3.6		2717.76		873.08		0.000
Cognitive survey	Or	3.45	0.000	1.18	0.000	0.000							
	Co	1.96		2.18		0.406							

PE: positive emotions; NE: negative emotions; Or: organic sample; Co: conventional sample; LC: light colors, DC: dark colors.
